# Chronic Venous Ulcers of the Leg

**Published:** 1986-02

**Authors:** S. J. Calder, D. J. Leaper

**Affiliations:** Medical Student; Consultant Senior Lecturer, University of Bristol, Department of Surgery


### Chronic venous leg ulcers and the role of dressings in their treatment

**S. J. Calder**
Medical Student,
**D. J. Leaper, MD, ChM, FRCS, FRCSEd**
Consultant Senior Lecturer, University of Bristol, Department of Surgery

### INTRODUCTION

About 0.5% of our population in the United Kingdom are estimated to suffer from chronic leg ulcers (1). In a study in Scotland ten patients per thousand of population were diagnosed as having this condition; one third starting before the age of fifty; the average length of time that ulcers persisted being fourteen years, with 3–4 episodes of ulceration. In the Bristol area there are approximately ten cases treated in each Health Centre; this being a prevalence rather than an incidence, which is more difficult to estimate.

There is a tendency to consider a chronic leg ulcer as a single entity when it is usually a sign of one of several underlying disorders. 85% are venous in origin, 10% are arterial, the remaining 5% being due to trauma, infection, neoplasm, artefact such as self-mutilation, pyoderma gangrenosum, vasculitis, and other rarities (2). In this review only chronic venous ulcers will be considered.

In most cases the clinical diagnosis of a venous ulcer is relatively straightforward. It is typically situated over the medial malleolus, oval or serpiginous in shape and shallow-muscle, fascia or deeper tissues are only rarely exposed (3, 4). The site varies from a few square millimetres to the whole ankle circumference (the gaiter ulcer). It is not usually painful, though associated degrees of oedema may cause pain. Surrounding skin changes of chronic venous insufficiency include hyperpigmentation, due to haemosiderin deposition, and hypostatic eczema—both of which usually precede the ulcer. Particular consideration must be given to features suggestive of other causes; especially the raised, rolled edge of neoplastic change. Biopsy of the edge is usually diagnostic.

The association of venous ulcers with varicose veins is not invariable. Venous 'stasis' ulcers are often incorrectly called varicose ulcers (5), despite the fact that it has been shown that patients with little or no sign of leg varicosities are equally likely to develop ulcers. Chronic ulceration is still a major primary end-point of venous (incompetent vein perforator valves) insufficiency (Table 1).

Table 1 illustrates how ulceration and varicosities are products of a mutual initiating pathology but are not mutually dependent. There is *no* causal relationship between them and neither is there a causal relationship between ulceration and thrombophlebitis (4).

**Table 1**

**Primary symptoms of venous insufficiency:**
[Raju, S(6)]

| | |
|---|---|
| Pain | 10% |
| Swelling | 21% |
| Ulcer | 53% |
| Discoloration | 2% |
| Varicosities | 14% |

### AETIOLOGY AND CURRENT TREATMENT OPTIONS FOR VENOUS ULCERS

This confusion of the exact causative pathology adds greatly to the clinical problems concerning choice of treatment. However, what is known is that a leg ulcer, once developed, represents full thickness skin loss with adverse factors to healing in addition to those found in, for example, a burn of comparable depth. These are (7): (a) the chronic history which implies a persistent underlying vascular disorder, (b) that over the medial malleolus wound contraction of skin edges is virtually impossible over tendons or joints. Therefore, re-epithelialization is the only way to restore the skin surface. This may be affected also by a skin graft which, if it 'takes' at all, provides at best a delicate cover over one of the most exposed and constantly traumatised parts of the body. Hence, the success of conservative treatment is dependent on its beneficial effect on the process of epithelialization and healing by secondary intention. Repair by this latter process provides better skin cover but re-epithelialization from the wound edges rarely extends beyond 1 cm.

So, in treating a venous leg ulcer both the surface defect and underlying cause must be attended to in an attempt to prevent recurrence which is inevitable unless some form of surgical intervention is considered (4). Surgery has two distinct roles to play in treatment—as a first line of attack in the form of skin grafting, or in obliteration of the cause of the venous insufficiency by ligation of incompetent veins (which we shall not discuss). For a split thickness skin graft to be successful, a radical excision of the underlying scar tissue may be required. Exposed periosteum or tendons, however, do not accept grafts. The sequence of events leading to this scarring is shown in Table 2.

**Table 2**

**Pathway for production of subcutaneous scarring in venous insufficiency**

perforator valves fail
blood flow from deep to superficial veins
raised blood pressure in capillaries
exudation of protein rich fluid, and small haemorrhages
raised tissue pressure
inhibition of blood flow in arterial capillaries
decreased oxygenation, and trophic skin changes, with ulceration in the long term, subcutaneous tissue is invaded by fibroblasts with perivascular fibrin cuffing
further skin thickening (lipodermato-sclerosis)
characteristic scar tissue up to 1.5 cm thick is laid down

This layer of scar tissue, lipodermato-sclerosis, beneath the ulcer bed justifies radical excision of the ulcer base before a graft:...'every surgeon who has cut through one (scar) will find it hard to believe that conservative treatment can lead to a lasting cure' (8). The majority of patients treated in this way achieve long-term relief from their chronic venous leg ulcers. If recurrence ensues or there is incomplete 'take' the ulcer will usually be far smaller and easier to manage, by further operative or conservative means (8).

Speed of healing is of the essence, since granulation tissue is gradually transformed to scar tissue, and the longer it takes before epithelium covers the defect, the thicker will be the scar.

Studies show (8) that when two parts of the same ulcer are treated, one with a graft, one healing spontaneously with conservative treatment, the grafted area recurs significantly less than the other. Skin is clearly the best dressing.

However, sheer numbers dictate that ulcers have to be treated conservatively in the community, in outpatients, or on long stay wards (which are also limited in number).

The mainstay of conservative treatment is the application of compression. Correct application of this is crucial; the skill being to achieve a graduated compression, with maximal pressure at the ankle. In practice, and proven by investigations (1), very few bindings approach this ideal. Elasticated bandages are preferable because they maintain a pressure over the primary dressing on the ulcer which would otherwise be compacted and lose its comprehensive role. Old bandages are not as efficient as new bandages; it has been shown that washing a bandage reduces its elasticity by nearly 50% (1).

Along with postural elevation, compression bandaging (with a non-adherent gauze such as Jelonet in immediate contact with the ulcer) will heal the majority of venous ulcers. If the ulcer shows signs of superficial colonising infection or slough on its surface, cleansing with Eusol or other antiseptic agents before applying the dressing is common practice. There is evidence that as well as cleaning and disinfecting the ulcer base, Eusol may damage healthy granulation tissue. Unpublished work and recent reports suggest that it may be more toxic to multiplying cells than was previously thought (1, 9). Eusol is however an ideal primary agent for debridement of excessive adherent slough but, once an ulcer is clean it should be replaced by a less toxic agent, such as Chlorhexidine, which has antimicrobial action without toxicity.

### THE IDEAL DRESSING

This toxicity contravenes the specifications for any topical agent used on ulcers; according to a BMJ editorial (10), an ulcer dressing should:

1. Mould to the wound contour.
2. Allow humidity and gas exchange.
3. Provide a stable, non-adherent contact layer—fibroblasts growing across a glass slide will grow at twice the speed if a glass cover is placed on top (7).
4. Have a high absorption mass superficially.
5. Not be incorporated into the wound unless biodegradable.
6. Not tear off new epithelium when removed, either as a result of cells growing into the dressing lattice, or becoming adherent on drying out.
7. Not contain an anti-infective agent which inhibits the formation of new cells.

In addition, the dressing should be water and bacteria impermeable, sterile, and thermally insulating (9).

Ulcer temperature should be maintained as close as possible to body temperature. Below 28°C phagocytic and mitotic activity are markedly reduced (9). Long exposure of wet wounds is a particular cause of heat loss. However, the thermal properties of secondary dressings (bandages or absorbent pads) can always make up for any lack in the primary dressing, so built-in insulating properties of primary dressings are really icing on the cake.

Before treatment, most ulcer bases are infected with pathogens to a greater or lesser degree in addition to colonising commensals such as Coryneiforms or Diphtheroids. This may give rise to unpleasant odour and soiling of dressings (11, 12). Most common infecting organisms are Pseudomonas aeruginosa, Staphylococcus aureus, Proteus; with B-haemolytic Streptococcus causing the worst delays in healing (13). Removal of Staphylococcus aureus from an ulcer is associated with significant increase in the healing rate (14). Other trials support these findings, but some ulcers heal satisfactorily despite heavy colonisation by Pseudomonas (14).

Trials of topical antibiotics used in ulcers have on the whole been inconclusive. Metronidazole, active against anaerobes which are relatively uncommon ulcer organisms given orally was claimed to help the healing of stasis ulcers (15), but analysis of the findings shows that the claims are excessive in the light of the results. Furthermore, a balance must be struck between the benefits of antibiotics (and at the moment there seems to be little place for them except given systemically for infective cellulitis), and their adverse effects of allergy and encouragement of resistant organisms; and of antiseptics with their toxic effects on the healing process. Most dressings do not have inherent anti-bacterial action, simple occlusive coverings such as 'Opsite', which contain the ulcer exudate, serve a useful secondary purpose because the exudate itself has considerable bactericidal properties (16).

### ULCER HEALING TRIALS

There is a lamentable dearth of clinical evidence to recommend any of the new products. Many quoted findings appear to be the results of case studies or in vitro experiments. There are reasons and certain hindering aspects to prevent scientifically acceptable and accurate trials (13, 17, 18):

(i) Patient selection; 'drop-outs' from a trial must be analysed and accounted for. Selecting reliable patients only, would incorporate a selectivity factor.

(ii) Objective methods of measuring ulcer size, such as cellulose acetate tracings or planimetry, give reasonably accurate and relatively reproducible measurements. Subjective assessments of the 'amount' of granulation tissue are only acceptable in very large samples when linear analogue scales are used.

(iii) The patients should be entered consecutively and randomly allocated to a treatment but such trials cannot be blinded in any way.

(iv) Optional cross-over from one treatment to another at a fixed time, often used in the comparison of two dressings, is not advisable due to difficulties in interpretation.

(v) Whether the trial should be multicentre or monocentre is debatable. A multicentre trial can be completed more quickly and is easier to generalise from, but the interpretation of treatment applications will vary between practitioners more than in a single centre study.

What is the best objective measurement of ulcer healing? There is clearly a need for a more scientifically acceptable and easily measured parameter. On one set of data for different sized ulcers, the percentage change in ulcer area was less biased than either (a) absolute change in ulcer area or (b) change in area divided by pre-treatment circumference (13). The shape of an ulcer influences the rate of healing; because it heals from the epithelial edge, the larger the circumference to area ratio, the more rapidly it should heal. Hence circular ulcers heal the slowest (13). In future trials group comparability may require to take ulcer shape into account.

The influence of duration of an ulcer is also significant. Statistical analysis concludes that 'the longer an ulcer has existed, the longer it will continue to exist' (13)—like many clinical problems! Perhaps these points go some way to explaining the lack of controlled clinical trials. A 1980 editorial (19) on ulcer dressings stated: 'What is needed are correctly controlled trials comparing this agent with some other...until the results of such a trial demonstrate unequivocally a significant benefit (biologically and clinically significant, not just a series of 'p' values below 0.05)...we shall continue to advise our doctors to divert resources to more useful enterprises.' This situation is equally applicable today.

### DRESSINGS AVAILABLE FOR CHRONIC LEG ULCER CARE

So what are the types of dressing available? The bewildering numbers, recently being increased severalfold, might suggest to the casual observer that none are totally satisfactory, and the scene is presently becoming something of an advertising battle-ground between the manufacturers.

Topical preparations which purport to encourage ulcer healing can be divided into three main groups (20):

1. Occlusive dressings, which can be either *simple*, such as Opsite, *medicated*, such as zinc impregnated bandages (Viscopaste and Calaband for example) or *complex*. The complex dressings depend on more specialized physical properties for their actions. Hydrogel dressings, such as the polyacrylamide Geliperm, have the ability to absorb and remove exudate across an osmotic gradient while remaining occlusive to harmful external influences.
2. Topical debriding and cleansing agents, such as Debrisan and Scherisorb or antiseptic-impregnated Iodosorb, which primarily remove slough and tissue debris from the ulcer base.
3. Impregnated dressings such as Jelonet or derivatives containing anti-microbials are more orthodox and commonly used as standard dressings.

All these dressings are used in combination with compression bandages with or without absorbent secondary dressings between. Table 3 shows a selection of dressings which are currently available.

Table 4 is a league-table which attempts to rate some of the various dressings, old and new on their relative merits. The results do not pretend to be accurate, but it is interesting to look at the dressings as objectively as possible and in relation to each other, because absolute data is non-existent. The information has been gleaned from:

(i) Manufacturers information sheets.
(ii) In vitro studies.
(iii) Case studies.
(iv) The few 'trials' published which have compared two or more of the dressings with a 'standard'.

By using a linear analogue scale, grading properties from 1 to 3, we have a feasible comparison between dressings. In the absence of satisfactory trials and the means to provide consistent quantitative data, we cannot be more definitive. Absolute values are of no use in comparing independent studies which all seem to produce positive findings. If nothing else, the Table shows that no single dressing is outstanding, but one may excel in a particular performance aspect. For example Iodo-

**Table 3**

**Description of the dressings**

| Dressing | Group | Presented as: |
|---|---|---|
| Opsite | simple occlusive | —transparent adhesive polyurethane film |
| Zinc Paste Bandages | medicated occlusive | —gauze bandage impregnated with zinc paste (many varieties available for many years) |
| Geliperm | complex occlusive | —transparent sheet of polyacrylamide hydrogel |
| Sorbsan | | —absorbent biogradable fibre weave derived from seaweed |
| Synthaderm | | —closed cell polyurethane foam sheet |
| Corethium | | —lyophilized porcine dermis sheet |
| Silastic foam | | —addition of a catalyst forms a tailored expanded elastomer foam dressing |
| Granuflex | | —flexible wafer—like hydroactive polymer that adheres to dry skin |
| Variclene | debriding | —green aqueous gel containing Brilliant Green and lactic acid |
| Iodosorb | | —microbeads of a starch polymer with iodine |
| Debrisan | | —spherical dextranomer beads |
| Scherisorb | | —starch hydrogel, in gel paste form |
| Aserbine | | —acid cream |
| Varidase | | —cream containing the enzymes streptokinase and streptodornase |
| Hioxyl | | —hydrogen peroxide cream |
| Bard Absorption Dressing | | —polysaccharide flakes reconstituted to form a gel paste |
| Jelonet | impregnated | —gauze impregnated with paraffin. Variants with other antimicrobial agents (antiseptics and antibiotics) |

sorb, Sorbsan and Bard Absorption dressing score well for their property to absorb excess exudate. Hioxyl cream is a superb cleansing agent, but unsuitable for use beyond the initial cleansing stages.

Because no dressing stands out as a 'complete' treatment regimen, nursing criteria for acceptance must also bear an important role: ease and frequency of dressing for example. The cost is a factor which will influence unit managers purse-strings. The hydrogels and complex debriding agents such as Debrisan, which is surprisingly widely used, are prohibitively expensive in comparison to Jelonet.

Finally, it is surely reasonable to take each ulcer individually, assess its stage of healing, its aetiology and its treatment needs (is it infected or covered by excessive slough?), whether it is possible to treat in the community, and select an appropriate dressing on this basis. As many of the newer dressings are expensive or not available by prescription few medical or nursing practitioners could allow themselves this luxury. Combined with a lack of education and interest in this field it is probably unlikely that ulcer care would advance even if complete freedom of choice of dressing were allowed. The core of the problem is that venous ulcers have been approached as if they were simply a defect in otherwise healthy epithelium; which they clearly are not. The underlying problems must be known and be recognised. In this light, a synthetic skin substitute is theoretically the answer. The hydrogels fulfil this role, but paraffin-gauze and simple dressings such as Melolin will continue to be used because of their cost effectiveness, until the results of a more scientific approach suggests otherwise.

If a science is a systematic and formulated knowledge, the use of leg ulcer dressings has yet to become a science.

### REFERENCES

1. (Cassette) Healing Venous Ulcers Medicassette Supplement, No 5. Sterling Winthrop 1985.
2. DAHLE, J. S. Conservative treatment of leg ulcers. In: Sundell, B. ed.; Symposium on Wound Healing, Espoo 1979, 143–151.
3. Anonymous: Diagnosis and treatment of venous ulceration. Editorial, Lancet 1982, ii; 247–248.
4. HARVINEN, P. Treatment of venous leg ulcers. In: Sundell B. ed; Symposium on Wound Healing, Espoo 1979; 135–140.
5. LOCKE, P. M., Riddle, M. D. The use of Synthaderm in the treatment of ischaemic leg ulcers. In: Sundell, B. ed; Symposium on Wound Healing, Espoo 1979; 135–140.
6. RAJU, S. Venous insufficiency of the lower limb and stasis ulceration. Ann Surg 1983 June 197(6); 688–697.
7. PEACOCK, E. E. Wound Repair. 3rd ed. W. B. Saunders 1984, 177–182.
8. ESKELAND, G. The surgical treatment of venous leg ulcers. In: Sundell, B. ed; Symposium on Wound Healing, Espoo 1979, 139–165.
9. WESTABY, S., ed. Wound Care. Nursing Times, 1981—Johnson and Johnson.
10. Anonymous. Wanted: a new wound dressing. Editorial B Med J 1979, II; 689.
11. LAWRENCE, J. C. Bacteriology and Wound Healing. In: Fox J. A., Fischer H. eds. Cadexomer Iodine Symposium, Schattauer Verlag, Stuttgart 1983; 19–34.
12. SCHELDERUP, H. Delayed wound healing caused by infection. In: Sundell, B. ed. Symposium on Wound Healing, Espoo 1979; 71–72.
13. FLYNN, M. J. Recent experiences with the design and interpretation of trials in leg ulcers. In: Fox, J. A., Fischer, H. eds; Cadexomer Iodine Symposium, Schattauer Verlag, Stuttgart 1983; 57–61.
14. SKOG, E. et al. A randomized trial comparing cadexomer iodine and standard treatment in the outpatient management of chronic venous ulcers. Br J Derm 1983; 109(1): 77–83.
15. Anonymous. Does metronidazole help leg ulcers and pressure sores? Drug Ther Bulletin 1982, 5; 20(3): 9–10.

*(References continued on page 11)*

**Table 4**

**References; 5, 9, 17, 20, 21, 22, 23, 24, 24, 26, 27, 28, 29, 30, 31, 32, 33, 34, 35, and Manufacturers Information**

### Chronic venous leg ulcers *(continued from page 9)*

16. LEAPER, D. J., Brennan, S. S., Simpson, R. A., Foster, M. E. Experimental infection and hydrogel dressings. J Hosp Inf, 5; 69–73.
17. ORMISTON, M. C., Seymour, M. T. J., Venn, G. E., Cohen, R. I., Fox, J. A. A randomized comparison of cadexomer iodine and a standard treatment in outpatients with chronic venous ulcers. In: Fox, J. A., Fischer, H., eds. Cadexomer Iodine Symposium. Schattauer Verlag, Stuttgart 1983; 63–69.
18. NEISS, A. Biostatistical aspects in planning and evaluation of a clinical trial of patients with ulcers cruris. In: Fox, J. A., Fischer, H. eds. Cadexomer Iodine Symposium. Schattauer Verlag, Stuttgart 1983; 71–73.
19. IVE, A., Cormaish, S. Topical therapy: leg ulcers. In: Rook, A., Savin, J. eds. Recent advances in Dermatology (5), 302-303. Churchill Livingston 1980, Edinburgh.
20. LEAPER, D. J., Stewart, A. Dressings for Ulcers. Unpublished data.
21. Young, H. L., Wheeler, M. H. Report of a prospective trial of Dextranomer beads (Debrisan) and silicone foam elastomer (Silastic) dressings in surgical wounds. B. J. Surg Vol 69 (1982): 33–34.
22. WALSH, J. A. Evaluation of Debrisan in the treatment of chronic leg ulcers. Med. J. Aus. 1980, 1; 373–375.
23. ALLEN, P. C., Turner, A. D. An evaluation of Debrisan in chronic leg ulcer and pressure sores. J. Int. Med. Res. (1979) 7; 459.
24. THOMPSON, I. L. Debrisan and leg ulcers. (Letter). Med. J. Aus. 1980, 1 (13); 263–265.
25. THORNE, N. Evaluation of Debrisan in the treatment of ulceration of the lower limb. Br. J. Clin. Prac. 1979, 33 (9); 263–265.
26. GROENEWALD, J. H. An evaluation of dextranomer as a cleansing agent in the treatment of the post-phlebitic stasis ulcer. S. African Med. J. 1980, 57; 809–815.
27. MYERS, J. A. Wound healing and the use of a modern surgical dressing (Opsite). Pharm. J., 31; 1982: 103–104.
28. ALVAREZ, O. M., Mertz, P. M., Eaglstein, W. H. The effect of occlusive dressings on collagen synthesis and re-epithlialization in superficial wounds. J. Surg. Res, 35; 142–148.
29. TURNER, T. D. Synthaderm—An environmental dressing. Pharm. J. 1982, 20.
30. LAWRENCE, J. C. The effect of synthaderm and other dressing materials on the bacterial content of experimental wounds. In: Sundell, B. ed. Symposium on Wound Healing, Espoo 1979; 109–115.
31. HANNUKSELA, M., Väätäinen, N. Synthaderm in leg ulcers—a preliminary report. In: Sundell, B. ed. Symposium on Wound Healing, Espoo 1979; 155–157.
32. ALLENBY, C. F., O'Callaghan, R., Vollum, D. I. Treatment of venous ulcers using Variclene. Br. J. Clin. Prac. 1983, Nov-Dec 37 (11–12): 382–384.
33. GUSTAVSON, B. Cadexomer iodine-introduction. In: Fox, Fischer, eds. Cadexomer Iodine Symposium, Schattauer Verlag, Stuttgart 1983; 35–39.
34. Anonymous. Porcine skin dressing. Drug & Ther. Bull. 1976; 14: 71–72.
35. NASAR, M. A., Morley, R. Lost effectiveness in treating deep pressure sores and ulcers Practitioner; 226:307–310.

